# Improving diets and nutrition through an integrated poultry value chain and nutrition intervention (SELEVER) in Burkina Faso: study protocol for a randomized trial

**DOI:** 10.1186/s13063-017-2156-4

**Published:** 2017-09-06

**Authors:** Aulo Gelli, Elodie Becquey, Rasmane Ganaba, Derek Headey, Melissa Hidrobo, Lieven Huybregts, Hans Verhoef, Romain Kenfack, Sita Zongouri, Hannah Guedenet

**Affiliations:** 1 International Food Policy Research Institute (IFPRI), 2033 K Street NW, Washington, DC 20006 USA; 2 AFRICSante, Bobo-Dioulasso, Burkina Faso; 3 London School of Hygiene & Tropical Medicine (LSHTM), London, UK; 4 ASI, Ouagadougou, Burkina Faso

**Keywords:** Impact evaluation, Diet, Nutrition, Poultry, Value-chain

## Abstract

**Background:**

The SELEVER study is designed to evaluate the impact of an integrated agriculture–nutrition package of interventions (including poultry value chain development, women’s empowerment activities, and a behavior change communications strategy to promote improved diets and feeding, care, and hygiene practices) on the diets, health, and nutritional status of women and children in Burkina Faso. This paper presents the rationale and study design.

**Methods:**

The impact evaluation involves a cluster randomized controlled trial design that will be implemented in 120 rural communities/villages within 60 communes supported by SELEVER in the Boucle de Mouhoun, Centre-Ouest, and Haut-Bassins regions of Burkina Faso. Communities will be randomly assigned to one of three treatment arms, including: (1) SELEVER intervention group; (2) SELEVER with an intensive WASH component; and (3) control group without intervention. Primary outcomes include the mean probability of adequacy of diets for women and children (aged 2–4 years at baseline), infant and young child feeding practices of caregivers of children aged 0–2 years, and household poultry production and sales. Intermediate outcomes along the agriculture and nutrition pathways will also be measured, including child nutrition status and development. The evaluation will follow a mixed-methods approach, including a panel of child-, household-, community-, and market-level surveys, and data collection points during post-harvest and lean seasons, as well as one year after implementation completion to examine sustainability.

**Discussion:**

To our knowledge, this study is the first to rigorously examine from a food systems perspective, the simultaneous impact of scaling-up nutrition-specific and nutrition-sensitive interventions through a livestock value-chain and community-intervention platform, across nutrition, health, and agriculture domains. The findings of this evaluation will provide evidence to support the design of market-based nutrition-sensitive interventions.

**Trial registration:**

ISRCTN registry, ISRCTN16686478. Registered on 2 December 2016.

**Electronic supplementary material:**

The online version of this article (doi:10.1186/s13063-017-2156-4) contains supplementary material, which is available to authorized users.

## Background

Optimal child nutrition and development is determined by dietary, behavioral, and health determinants, influenced by underlying food security, caregiving resource, and environmental conditions [1]. Low-income households typically subsist on monotonous staple-based diets and lack of diet diversity is associated with inadequate intake and risks of deficiencies of essential micronutrients [2, 3]. Food production is just one factor in the availability and consumption of nutrients [4]. Food is stored, distributed, processed, marketed, prepared, and consumed in ways that affect the access, acceptability, and nutritional quality of foods for consumers during critical stages of the life cycle, including infancy, childhood, and adolescence.

An emerging framework for improving diet quality involves value chains (VC) [5]. The VC framework focuses on the broader range of actors that are involved in the production, processing, trade, and consumption of food products and the opportunities to achieve beneficial outcomes for some or all of the actors through changes in the structures, systems, and relationships that the chain involves. However, VC analysis and development have historically not considered nutrition-related value-addition; rather, the focus has been mainly on increased income for VC actors. Because VCs play a key role in determining food availability, affordability, quality, and acceptability, they also have a role in improving diets [6] and, moreover, provide useful lenses to characterize the food system. Participation in VCs in low- and middle-income settings also carries important opportunities to expand benefits to women by increasing women’s assets, skills, and decision-making power within both the household and the community [7]. At the same time, agricultural development, especially related to expansion and formalization of markets, may also inadvertently disempower women, by adding to their time burden and/or reducing their control over income, which could have negative consequences for diet and nutrition outcomes [7].

VC-related activities may also increase health risks in producers and consumers. One specific emerging risk with livestock keeping is the elevation of disease burdens related to the close proximity of animals and humans, which include increased exposure to zoonotic diseases, including elevated risks of diarrhea [8] and environmental enteric dysfunction (EED), [9] as well as respiratory infections [10]. Poultry are a particular concern, given that scavenging poultry systems involve poultry roaming in and around the main household dwelling, thereby exposing young children (who are often left alone to sit or play on the ground) to the ingestion of chicken fecal matter [9]. Evidence on these risks and exposures is, however, limited to a small number of observational studies. EED is thought to reduce appetite, inhibit nutrient absorption, impair immune system function, and is associated with stunting in children [11]. To date, the prospects of achieving improved diets (especially of women and young children) through interventions in livestock/poultry VCs have been largely ignored and little attention has been paid in these VC interventions to the prevention of EED through improved poultry-related hygiene promotion. Likewise, water, sanitation, and hygiene (WASH) interventions have largely focused on reducing exposure to human fecal matter, even though animal feces are more widespread in typical village environments [12]. There are several reasons for this evidence gap, including the issue that the biological processes involved in EED are complex and not yet well understood [11]. Moreover, examining the links between poultry husbandry and child nutrition also requires researchers to design rigorous research across traditional disciplines which is in itself a challenge.

### Country context

Burkina Faso is one of the least developed countries in the world, with the latest Demographic Health Surveys [13] showing high rates of child stunting (35%), wasting (16%), and anemia (88%). Almost half of women are anemic and 16% of women are underweight (BMI < 18.5 kg/m^2^). Infant and young child feeding (IYCF) practices are particularly poor. Exclusive breastfeeding up to six months occurs in less than 13% of infants and only 3% of children are introduced to solid or semi-solid foods in the six- to eight-month window [13]. Particularly pertinent for our study is the low rate of dietary diversification for young children (age range 6–24 months). In an international sample of recent DHS data, we estimated that Burkina Faso had the second lowest dietary diversity score in the world (based on a count of seven food groups) [14]. In the 2010 DHS, 14% of children aged under two years had consumed poultry flesh and egg consumption was limited to 3% of children in the same age group, while 80% of households owned poultry. These findings suggest that there is significant scope to further expand poultry-based income generation for rural households, and chicken and egg consumption for young children and their mothers. Despite woman’s crucial role in poultry production in Burkina Faso, women generally have limited control over the mobility of their poultry to markets, often relying on husbands and sons to take poultry to market and connect to more formal traders [15]. Moreover, a gender gap in poultry ownership and access to services exists with women owning smaller flocks and having less access to veterinary services [15].

### The SELEVER intervention

Soutenir l’Exploitation Familiale pour Lancer l’Élevage des Volailles et Valoriser l’Économie Rurale (SELEVER) or Women’s Poultry Program to Improve Income and Nutrition project is a five-year program funded by the Bill & Melinda Gates Foundation. The SELEVER project is implemented by Agribusiness Systems International (ASI) in partnership with selected local NGOs, private institutions, and governmental services. SELEVER is a poultry VC program that leverages agriculture development strategies and nutrition to increase poultry production and improve the nutritional status of women and children in the Centre Ouest, Hauts-Bassins, and Boucle de Mouhoun regions of Burkina Faso. The project uses an integrated approach combining revenue generation, women’s empowerment, and nutritional behavior change interventions, including:

- **Improved access to VC services.** This component includes vaccinations, financing, and training on poultry flock management (including housing). SELEVER will strengthen the capacity of village level volunteers to provide vaccination services for poultry producers, aiming to increase vaccination against Newcastle’s disease, reduce poultry mortality, and increase poultry production and revenues. The commercial relations between village volunteer vaccinators (VVVs), other chain actors, and service providers will also be supported to enhance the efficiency of the poultry VC as a whole. In addition, the SELEVER training package for VVVs will include activities around nutrition-related behaviors, including consumption of animal source foods and basic hygiene practices.
- **Behavior change communication (BCC) on nutrition and health.** This will be provided through women’s groups, poultry producer groups, and local community leaders. The content of the BCC activities includes the promotion of improved diets at key stages of the life cycle, including IYCF practices (including breastfeeding promotion for infants) and basic hygiene.
- **Community-level sensitization on women’s economic empowerment and gender equity, including strengthening of women’s groups.** The activities under this component include training participants from existing women’s associations on enterprise development, including village saving and loans and enhancing commercial opportunities. The activities will also focus on strengthening women’s role in decision-making within households and the community on entrepreneurship, nutritious food production, marketing, consumption, and in child health, feeding, and care.

The SELEVER activities will be implemented through a training-of-trainers model. Local NGOs field staff will train producer group volunteers, VVVs, and women’s association volunteers who will then train their group members once a month or deliver key messages to households. ASI is planning training sessions with men and local leaders as well as a communications campaigns with radio spots and theatre troupes.

#### The enhanced WASH component

In addition to the SELEVER package, a more intensive hygiene component (SELEVER+) is being designed to improve health and hygiene in the context of poultry flock management that will be implemented as part of a sub-study on WASH. The WASH component will include BCC based on community-led total sanitation and baby-WASH concepts, with specific messaging tailored to also address hygiene-related constraints involved in poultry rearing.

### Study objectives

This study is aimed at evaluating the impact and costs of an integrated package of nutrition and agriculture interventions on the diets, health, and nutritional status of women and children in Burkina Faso. By also adopting a theory-based approach to the evaluation, measuring changes across a range of intermediate outcomes on the agriculture nutrition pathways (including poultry production, consumption, and the associated health environment) we will provide rigorous evidence on the effectiveness and trade-offs involved.

The main research questions for the evaluation include:

1. What is the impact of the SELEVER integrated agriculture–nutrition package (including VC development, gender empowerment, and BCC related to food intake, care, and hygiene practices) on the diets and nutrition status of women and children and on children’s morbidity symptoms?
2. Does incorporating a comprehensive poultry hygiene component to the agriculture–nutrition package have an additional impact on children’s nutritional status and morbidity symptoms?
3. What are the main factors and trade-offs that mediate the impact of the agriculture–nutrition package on women and children?
4. What are the impacts of a gender-sensitive agriculture–nutrition package on women’s empowerment and decision-making?

## Methods

### Program theory

The program theory for this evaluation is guided by the 2013 Lancet Series framework on Maternal and Child Nutrition [1]. The SELEVER package bundles a nutrition-specific intervention (BCC on nutrition, health, and care) alongside two nutrition-sensitive interventions (women’s empowerment and poultry VC development). The premise for understanding the role of VCs (that are, by definition, commodity-specific) in improving nutrition is that, generally, food-related nutritional impacts derive from changes in quality of overall diet, not just nutrient content of an individual food. Improvements in diet quality may also be necessary but not sufficient to result in improved nutrition if, for example, limiting constraints in the other determinants of nutrition (namely low burden of infectious diseases and caregiving practices) are not also simultaneously addressed. We identify four interlinked, food system-based pathways through which the SELEVER package could have an impact on diets. Building on [5] and [6], these pathways are based on: (1) leveraging demand; (2) supply of nutritious foods; (3) enhancing nutrition related value addition along a chain; and (4) empowering women. These pathways are complex, span multiple domains, and are linked through interactions with the food environment, including food availability, affordability, acceptability, nutrient quality, and food safety.

#### Pathway 1: increasing the demand for nutritious foods

Increased intake of nutritious foods through SELEVER can be achieved through the BCC promoting the consumption of, or willingness to pay for, nutritious food. Nutritious food may be produced by the same households consuming the food or may be purchased. Intake of the poultry VC-related food (eggs, chicken meat) could complement or substitute the consumption of other foods in the diet. The food may also be shared within the household or consumed by only a few household members. This pathway can also influence VC actors since greater demand for nutritious foods can lead to expanding marketing opportunities (see Pathway 2). Moreover, increased demand can play an important role in stimulating agricultural production, particularly for smallholders who face market-access constraints. The extent of the effect of the increased demand on prices will depend on the level of market integration. In addition, by improving knowledge on child health and care practices, BCC can also influence the other two immediate determinants of child malnutrition and lead to improved nutrition status of children in the first 1000-day window.

#### Pathway 2: increasing the supply of nutritious foods

This involves the more recognizable VC pathway, where interventions target chain actors in the upstream segment of a VC (e.g. producers) who often face multiple constraints in responding to demand from actors further downstream (e.g. retailers, processors). Interventions would look to alleviate these constraints (e.g. increase poultry flock size through reduced mortality from improved uptake of vaccinations and other poultry management practices), strengthening market channels while increasing production, reducing transaction costs and risk, leading to increased efficiency and profits, and, in time, to improved incomes. In addition to the increased supply of foods, increased production and incomes for smallholders could mean that some additional income feeds back into dietary decisions, further increasing consumption and demand for nutritious foods (see Pathway 1). SELEVER activities aim to increase poultry production through input provision and/or training on improved management practices, including the promotion of improved production technologies (housing, vaccinations) and increasing access to credit to stimulate investment in production. These VC interventions can also influence the basket of products (e.g. foods for local consumption rather than export and foods with relatively high nutritional value) that households produce. The targeting of particular foods has to be undertaken in the context of the substitution between crops for production and consumption and the long-term impacts for both incomes and nutrition at multiple scales (from the household and village levels to regional or national levels).

#### Pathway 3: value chain performance and nutrition-related value addition

The performance of the market-based transactions along the VC affects commercial relations and profits for the actors involved. In addition to economic value, the nutrition content, food safety, or contamination risk of a particular food can also be enhanced or diminished at key points along a VC. Moreover, by influencing supply volumes (quantity), price, and quality (including nutrition content and food safety/contamination) of a relevant food, VC performance can also mediate the effects of the SELEVER package on consumption and production decisions, influencing diets and nutrition of a broad range of target populations. These effects will be both direct (e.g. on poultry producers through the provision of VC services) and indirect (e.g. on consumers through market availability and prices). However, for nutrition-related value-addition to factor in investment strategies and in the VC transactions requires reliable information on both nutrition content and contamination risk to be transmitted along the chain and for prices to reflect a premium for these qualities. In practice, this is often not the case, particularly in low-income settings. As nutrition-related value includes properties that are akin to those of credence goods, there are no incentives to pay for quality unless there is some form of visible, third-part activity that may be undertaken publicly (e.g. information campaigns) or privately (e.g. consumer reporting). One of the VC-related contamination risks we will examine explicitly is related to the effect of livestock VC production practices on disease burdens, particularly through children’s exposure to animal feces.

#### Pathway 4: empowering women

As women play important roles in production and value addition, agriculture has the potential to empower women to make better food-, health-, and care-related decisions for themselves and their families [7]. Participation in VCs carries important opportunities to expand benefits to women by increasing women’s assets, skills, and decision-making power within both the household and the community [7]. At the same time, agricultural development, especially related to expansion and formalization of markets, also has the power to inadvertently disempower women, by adding to their time burden and/or reducing their control over income, which could have negative consequences for diet and nutrition outcomes. Empowering women is an integral part of the conceptual design of SELEVER. In particular, SELEVER aims to increase women’s income from poultry and improve their decision-making within the household. To this end, SELEVER will engage with female producer groups and savings and loans groups, and improve women’s access to saving and credit. SELEVER will, at the same time, engage village leaders and men in gender sensitization.

### Main hypotheses and outcome indicators

The SELEVER package is expected to have direct and indirect effects on a range of different target groups (Table 1).

**Table 1:** Summary of hypothesized impact pathways for SELEVER

		Expected effects	
SELEVER activity	Target group	*Proximal*	*Distal*
BCC on nutrition, diets, and IYCF practices	Producers, consumers	Changes in knowledge of nutritious foods in women and caregivers of children aged < 5 years	Changes in intake of nutritious foods and overall diets in women and children aged < 5 years
		Changes in knowledge on nutrition, health, and care practices among mothers of children aged < 2 years	Changes in child health, care, and feeding practices among mothers of children aged < 2 years
Strengthening poultry VC services	Producers, other VC actors, consumers	Changes in poultry production practices in poultry producer households	Changes in output and revenues in poultry producer households
		Changes in market opportunities and risk in poultry producer households	Changes in production systems in poultry producer households
		Changes in VC practices (including hygiene) in actors along the poultry VC	Changes in margins and costs in actors along the poultry VC
			Changes in hygiene and health environment in poultry producer households
Sensitization on women empowerment	Producers, consumers, other VC actors	Changes in women’s empowerment (e.g. time use and decision-making)	Changes in women’s wellbeing

The SELEVER package of interventions is expected to have a positive effect on:

- Diets of women and children aged < 5 years by increasing caregiver knowledge on nutritious foods, influencing food purchase and production decisions, benefitting both poultry producers and other households receiving the BCC in the intervention villages.
- Infant and young children’s nutrition and child-caring practices by increasing caregiver knowledge on such practices, benefitting poultry producer and other households receiving the BCC in the intervention villages.
- Poultry production practices, inputs, outputs, and revenue of smallholder poultry producers in the intervention villages. Other households may benefit directly or indirectly via increases in economic opportunities in the intervention villages.
- Sales and revenues of VVVs providing vaccination and other VC services in the intervention villages.
- Women’s empowerment by increasing women’s participation in VC activities in the intervention villages.

In addition, the SELEVER+ WASH arm is expected to have a positive effect on:

- Household hygiene and health environment leading to improvements in child infection and morbidity rates.

### Study outcomes

The primary outcomes of the study are focused on diet quality measures including the probability of adequacy (PA) for iron, zinc, and vitamin A, and mean probability of adequacy (MPA) in micronutrient intake for women (aged 15–35 years at baseline) and children (aged 2–4 years at baseline). Other main study outcomes include dietary diversity, minimum acceptable diet (for children aged 6–23 months), and household poultry production, sales, and profits. The program effects will also be assessed on several secondary outcomes and intermediate indicators identified through the program impact pathway analysis (see “Data collection and outcome measurement”).

### Design of the impact evaluation

The impact evaluation involves a cluster randomized controlled trial design that will be implemented in 120 rural villages within 60 communes supported by SELEVER in the Boucle de Mouhoun, Centre-Ouest, and Haut-Bassins regions of Burkina Faso.

### Study site

The intervention is targeted to rural and peri-urban communities within the three targeted regions. The geographical area for intervention was targeted by ASI on the basis of a set of variables related to poultry production and market access.

### Study population

The two primary reference groups for this study include women aged 15–35 years at baseline with at least one child aged 2–4 years at baseline and their (index) child aged 2–4 years, in the 120 villages targeted by the study. The secondary reference age group includes the siblings of the index children aged < 5 years who live in the same households as the index children.

#### Inclusion criteria

- Women aged 15–35 years with at least one (index) child aged 2–4 years living in the same household in the targeted villages
- Children aged 0–5 years at baseline whose mothers and index sibling are included in the study
- Households of women included in the study

#### Exclusion criteria

- Household head, child, parent, or guardian unwilling to participate in the study
- Households planning to move out of the study area within six months after the baseline survey
- Households were located in villages that were too small to have 15 or more households with children in the 2–4-year age group based on latest DHS demographics or too large to be considered rural (with population over 5000 people (or over 95% of the population distribution)

### Random assignment and manipulation of treatments

Communities will be randomly assigned to one of three treatment arms:

1. SELEVER intervention group: communities where the SELEVER program is implemented;
2. SELEVER+: communities where the SELEVER program is implemented alongside an intensive WASH component;
3. Control group: communities with no intervention, for at least the four years of the study duration.

The SELEVER intervention is implemented at the commune level as the training, sensitization, and BCC involve commune level stakeholders. During the preparation stage, 60 communes were selected from a pool of 79 communes that were available for scale-up by SELEVER in the targeted regions. The criteria for commune selection included: (1) not included in the SELEVER pilot communes; (2) classified as rural or peri-urban (as classified in the national census); (3) year-round accessibility by road; and (4) for the Hauts-Bassin region communes, proximity to the Boucle de Mouhoun and Centre Ouest regions. The first stage of randomization was aimed at randomizing the 60 communes in the study into two groups (SELEVER and control) and provides the basis for the main trial comparisons (SELEVER – Control). Due to the relatively small number of clusters in the study, to ensure balance in the comparison groups the random allocation was undertaken through restricted randomization by modeling selection using a set of commune-level and village-level variables obtained through the national census of 2006.

**Fig. 1:**
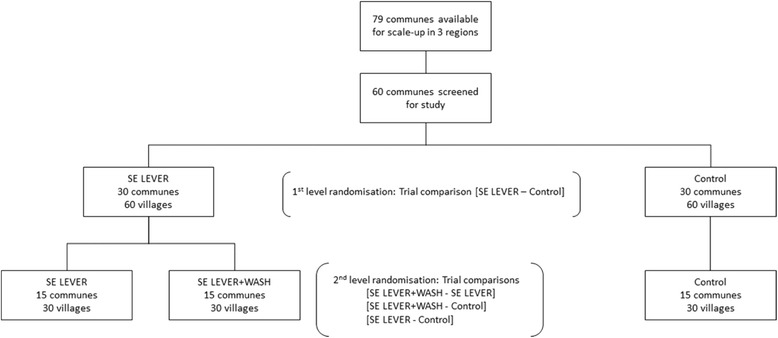
*Schematic view* of the randomization

**Fig. 2:**
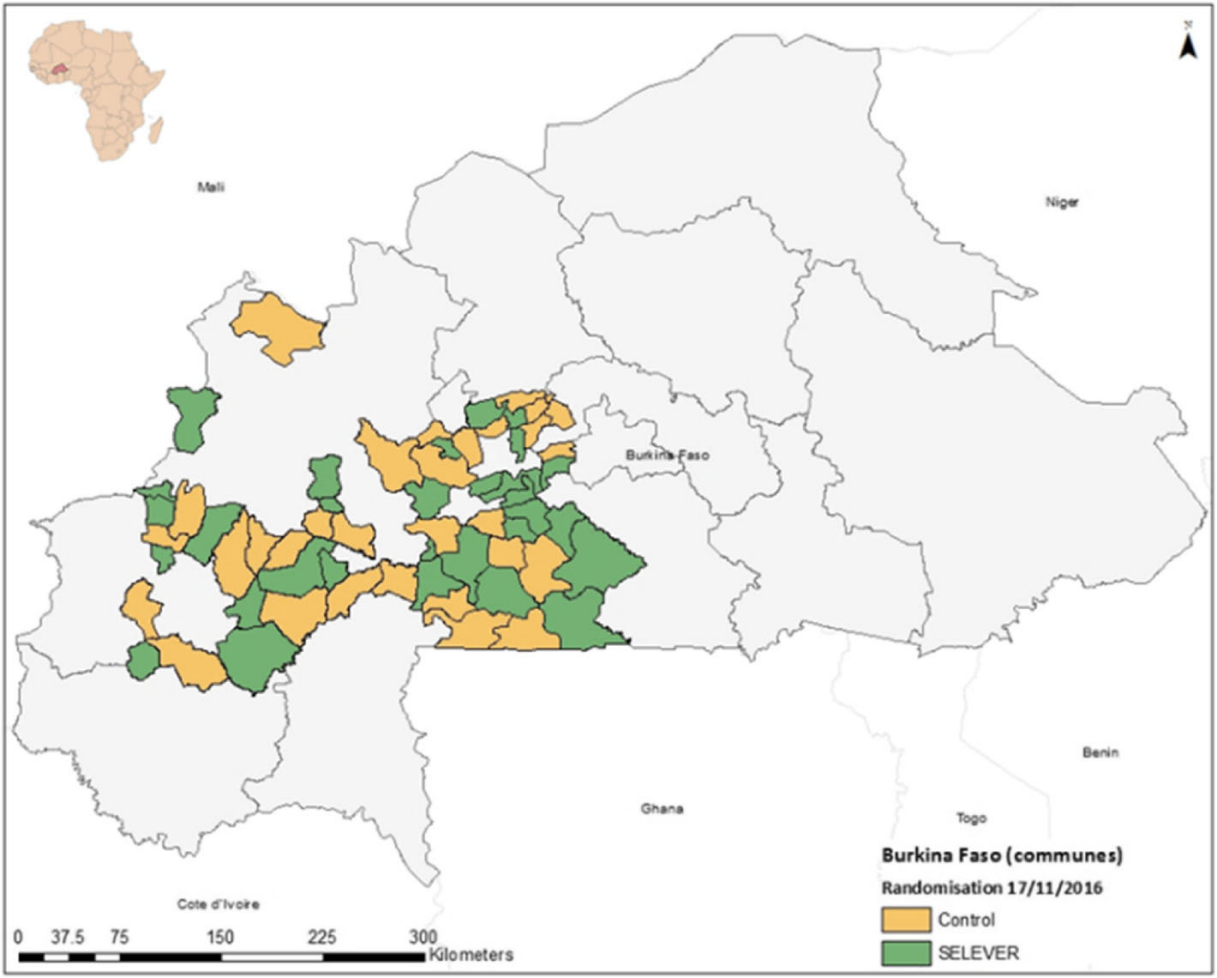
Map of study communes and first stage randomization

Following Hayes and Moulton [16], the variables in the restricted randomization were selected on availability and their potential influence on the main study outcomes. The variables in the model included population size, existence of a government center, number of female associations, main agricultural crop, main source of revenue, market presence, health center presence, number of functional boreholes, and number of functional wells. Village level data were also used to generate commune level aggregates for population size, number of health centers, number of boreholes, and market access. Villages that were too small to allow for a subsequent survey sample to be drawn (likely to have less than 15 households with children in the 2–4-year age group based on latest DHS demographics) or too large to be considered rural (with a population of over 5000 people or over 95% of the population distribution) were excluded from the list. A program was developed using Stata to randomly allocate communes to two different groups stratifying by region and then select two villages in each commune from the list of available villages. The algorithm then regressed the selection into the treatment group based on the village-level and commune-level covariates. The routine tested 3000 random allocations and selected the permutation that minimized the r^2^ statistic for the predicted selection. The second stage of randomization will provide the basis for the WASH sub-study comparisons (SELEVER+/SELEVER – Control). The 30 SELEVER village pairs (one pair for each commune) will be randomly assigned to either the SELEVER intervention or SELEVER+, and 15 communes from the control group will also be selected for the sub-study using a similar restricted randomization procedure (Fig. 1 and 2).

### Sample sizes

Power calculations (Table 2) and resource availability suggested the adoption of a dual sampling strategy: For the outcomes in the main trial comparison (SELEVER vs. control), 15 households from each of the 120 villages in the study will be surveyed at baseline and end line surveys, for a total of 1800 households. A sub-sample of 1080 households, women and children dyads (or pairs) will be included for detailed and more frequent assessments (see “Data collection and outcome measurement”).

**Table 2:** Minimum detectable effect size estimates and summary of parameters used in sample size calculations for the main study outcomes^a^

	Women’s dietary MPA	Children’s dietary MPA (3–5 years)	Children DD (6–24 months) (n. of food groups)	Household poultry production	Household poultry sales
Communes (n per intervention arm)	15	15	30	30	30
Villages (n per intervention arm)	30	30	60	60	60
Individuals per village (n)	12	12	8.8	15	15
Intra-cluster correlation (level 3)	0.002	0.002	0.001	0.001	0.001
Intra-cluster correlation (level 2)	0.05	0.04	0.05	0.06	0.01
Sample size (per intervention arm)	360	360	526	900	900
Estimated baseline levels	0.26	0.43	3.95	11	1
Standard deviation (baseline)	0.22	0.31	0.97	11	3
Minimum detectable effect size (SD)	0.28	0.27	0.21	0.18	0.15
Minimum detectable effect size (eq. levels)	0.06	0.08	0.20	1.98	0.45

^a^Estimates based on type I error of 5% (two-sided test) and statistical power of 80%

*MPA* mean probability of adequacy, *DD* dietary diversity

During the baseline survey, a household census will be conducted to identify all the large poultry producers in the community, households with women in the target groups (women aged 15–35 years with children in the target age group), and collect other basic household level information necessary for the sample selection. Households will then be randomly selected from the household census for the survey interviews. Large poultry-producing households (e.g. defined as owning a poultry flock of over 20 chickens/fowls) will be oversampled (6/15 households will be large poultry-producing households). This distribution of the sample will enable the construction of comparable samples for the main trial comparisons, also allowing for analysis of direct and indirect effects by including both beneficiary and non-beneficiary households in the survey population.

For the power calculations, we used data from an observational study examining food intake in two of the three regions of interest (Centre-Ouest and Boucle du Mouhoun) [17, 18] and from the impact evaluation of the Hellen Keller International homestead food production intervention in Burkina Faso [19]. The power calculations for the primary outcomes for women, children, and households are based on 80% statistical power and alpha value of 0.05 (Table 2). With a random sub-sample of 360 women and children per treatment arm in the detailed biomedical assessment, we can estimate effect sizes for the primary outcomes of about 0.28 z-scores in the MPA for women and children. This effect size is equivalent to about 6 points in MPA, which is approximately half the absolute difference observed between MPAs in the post-harvest and lean seasons in the targeted regions of Burkina Faso [17]. Considering that dietary assessment is quite time-consuming in terms of both data collection and analysis, and that biomarker status assessment is invasive and quite expensive, it is appropriate to limit assessment of these indicators to the required sub-sample only. The individual PA for a specific nutrient is compiled using the probability approach to compare the usual nutrient intake to the relevant population requirement distribution [20]. Therefore, its average at the population level can be interpreted as the prevalence of adequacy of intake for this specific nutrient. The MPA is an aggregate measure of the adequacy of intake across a range of key nutrients, in this case iron, zinc, and vitamin A. The other primary outcomes (e.g. dietary diversity, household poultry production, and sales) will require a larger sample size to detect a meaningful impact. With the full sample based on 900 households per treatment arm in the main trial (SELEVER – Control), we can detect effect sizes ranging from 0.18 z-scores (e.g. household poultry production) to 0.15 z-scores (e.g. household poultry sales). Power estimates for the study’s end line survey will be reassessed based on actual levels and intracluster correlations in our survey and estimated typical level of attrition based on similar study designs (set at 5%).

### Data collection and outcome measurement

**Fig. 3:**
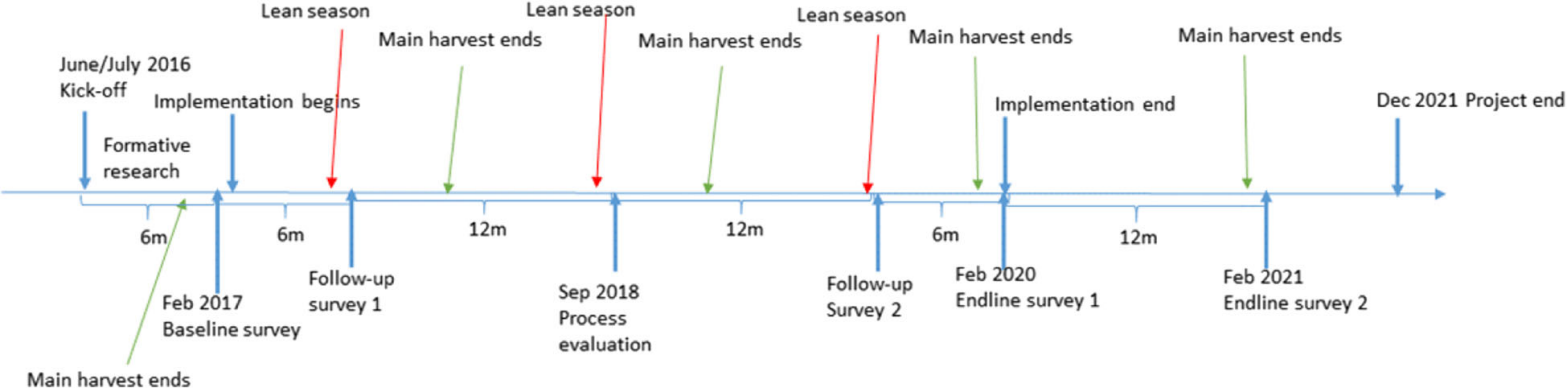
Stylized high-level data collection timeline

The evaluation will follow a mixed-methods approach with a panel of repeated measures (Fig. 3). Post baseline, follow-up survey 1 (six months after baseline), will examine the panel for seasonality and short-term changes across the different treatment arms. A process evaluation will be undertaken 18 months after baseline to provide data for minor adjustments to the SELEVER design. Follow-up survey 2 will provide evidence on two-year impacts during the lean season, while end line survey 1 will provide evidence on the impact of SELEVER after three years of implementation. End line survey 2 will provide evidence on sustained effects one year after SELEVER completed implementation. The panel design with repeated measures will also allow studying the intervention effects on the growth and nutritional status of children from birth throughout early childhood. The data collection includes child-, caregiver-, household-, and village-level data collection and will be undertaken using electronic tablets (Table 3).

**Table 3:** Measurement schedule for key study indicators

*Main trial comparisons*			Survey round				
Longitudinal, 1800 households			BL (Feb 2017)	R2 (Aug 2017)	R3 (Aug 2019)	R4 (Feb 2020)	R5 (Feb 2021)
Primary	Dietary intake	Dietary diversity (women and children)	I			I	I
	IYCF practices	Minimum acceptable diet (6–24 months)	I			I	I
	Poultry output	Production, sales, and profits	I			I	I
Secondary	Women’s empowerment	WEAI	I			I	I
	Maternal stress and wellbeing		I			I	I
	Poultry VC		I			I	I
	Morbidity	Recall of diarrhea, fever, and chest infections	I			I	I
	Hygiene	Exposure to poultry feces and cleanliness	O			O	O
		Hygiene knowledge	I			I	I
	Women’s nutrition knowledge	Nutrition KAP	I			I	I
	Nutrition	Anthropometry	M			M	M
	Expenditures		I			I	I
	Other household and maternal factors		I			I	I
*WASH sub-study comparisons*			Survey round				
Longitudinal, 1080 households			BL (Feb 2017)	R2 (Aug 2017)	R3 (Aug 2019)	R4 (Feb 2020)	R5 (Feb 2021)
Primary	Dietary intake	Probability of adequacy (Fe, Zn, Vit A), IDDS	I	I	I	I	I
	IYCF practices	Minimum acceptable diet (6–24 months)	I	I	I	I	I
	Poultry output	Production, sales, and profits	I	I	I	I	I
Secondary	Maternal stress and wellbeing		I	I	I	I	I
	Poultry VC		I	I	I	I	I
	Morbidity	Recall of diarrhea, fever, and chest infections	I	I	I	I	I
	Hygiene	Exposure to poultry feces and cleanliness	O	O	O	O	O
		Hygiene knowledge	I	I	I	I	I
	Women’s nutrition knowledge	Nutrition KAP	I	I	I	I	I
	Nutrition	Anthropometry	M	M	M	M	M
	Expenditures		I	I	I	I	I
	Other household and maternal factors		I	I	I	I	I
	Micronutrient status (children)	Hemoglobin (Hb)	B			B	B
		Plasma ferritin (PF)	B			B	B
		Soluble transferrin receptor (sTfR)	B			B	B
		Retinol-binding protein (RBP)	B			B	B
	Infection (children)	Serum C-reactive protein (CRP)	B			B	B
		α-1-acid glycoprotein (AGP)	B			B	B
		Malaria	B			B	B
		Pathogenic gut bacteria	S			S	S
	Cognition (children)		T			T	T

*B* blood, *BL* baseline, *I* interview, *IDDS* individual dietary diversity score, *IYCF* infant and young child feeding, *KAP* knowledge attitude practices, *M* measurement, *O* observation, *R* follow-up round, *S* stool, *T* test, *WEAI* Women’s Empowerment in Agriculture Index

#### Dietary assessment and anthropometry measurements

Within each survey household, basic anthropometric data collection will include all women of childbearing age (aged 15–35 years), all children aged < 5 years at baseline, and all children born within the households during the years between the baseline and end line surveys. A sub-sample of index women and children dyads (pairs) will be randomly selected for in-depth assessment, including a dietary assessment and biomedical tests. Dietary assessment will be undertaken by trained and supervised enumerators using the interactive 24-h recall multiple-pass method at each survey round [21]. The recall will be repeated at each round on a non-consecutive day on 15–20% of the sub-sample for the calculation of usual nutrient intakes. Prior to the recall interview, caregivers will be briefed on the purpose and methods of interview. The interview will be conducted using visual aids to assist in estimating portion sizes of the foods consumed. A short food frequency questionnaire assessing the frequency of consumption of more nutrient dense foods like poultry and other meat products over the last month will be included in the 24-h recalls. The child’s weight will be recorded using an electronic scale (SECA 876, Germany) to the nearest 100 g. Height or length will be measured to the nearest 0.1 cm using portable fixed base stadiometers or length boards (SECA 417, Germany). Mid-upper arm circumference (MUAC) will be recorded by using non-stretchable tape with 0.1 cm precision (SECA 201, Germany). All measurements will be taken in duplicate by an anthropometrist and an assistant. All measurements will be exercised before the study through standardization exercises. From these standardization sessions, inter-observer and intra-observer variation of measurement error will be documented. Maternal MUAC, weight, and height will also be recorded using non-stretchable tape (SECA 201), scales (SECA 877), and stadiometers (SECA 217, Germany), respectively. WHZ and HAZ scores will be calculated using the 2006 WHO growth standards [22]. IYCF knowledge and practices of caregivers will be assessed using a structured questionnaire based on the WHO guidelines [22].

#### Assessments of micronutrient and infection status

Blood and stool samples will be collected at baseline and year 3 and 4 end lines as part of the WASH sub-study. Infection biomarkers in the women and children dyads include serum C-reactive protein (CRP) and α-1-acid glycoprotein (AGP). Additional indicators on the presence of specific bacterial gut infections in stools will also be included in children. The potential list of pathogenic bacteria includes *Salmonella* spp., *E. coli*, enteropathogenic *E. coli* (EPEC), enterohemorrhagic *E. coli* (EHEC), *E. coli* genotypes associated with resistance to β-lactam antibiotics (extended spectrum beta-lactamase [ESBL]), *Staphyococcus aureus*, *Giardia intestinalis*, *Campylobacter jejuni*, methicillin-resistant *S. aureus* (MRSA). EED biomarkers in stools will include alpha-1-antitrypsin (AAT), neopterin (NEO), and myeloperoxidase (MPO). A heel or finger prick blood drop will be used to measure hemoglobin concentrations (using a Hemocue haemoglobinometer) and *Plasmodium* infection (using rapid diagnostic tests). An additional 300 μL of blood will be collected in lithium-heparin-coated microvettes (CB 300; Sarstedt) for measurement of retinol-binding protein (RBP), plasma ferritin (PF), transferrin receptor (TfR), CRP, and AGP concentrations. Immediately after phlebotomy, pre-labeled microvettes will be placed in an ice chest/cold box with ice packs, maintained at 0–8 °C for transportation to a central laboratory. Transport of the blood samples from the field will be on a daily basis and the samples will be processed no later than 24 h after phlebotomy. Precautions will be taken to maintain proper temperature and to avoid physical shocks in order to prevent hemolysis of blood cells during transportation. Upon arrival at the central laboratory, the blood samples will be centrifuged for plasma separation. For analysis, 100 uL of plasma will be aliquoted. The remaining plasma will be stored separately as a back-up. Pre-printed labels having identical numbers will be provided to the technicians at the central laboratory for use on the aliquots containing plasma before placing them in the freezer. The samples will be temporarily stored in a freezer at – 22 °C in the central laboratory. The aliquots will be shipped on dry ice to VitA-Fe Tech laboratories (Willstaett, Germany) for the micronutrient and inflammation marker analyses by enzyme-linked immunoassay [23].

#### Women’s empowerment and wellbeing

Women’s empowerment will be measured over the five domains of the Women’s Empowerment in Agriculture Index (WEAI), which include decisions about agricultural production, access to and decision-making power over productive resources, access to financial resources, leadership in the community, and time use. For this study, we will be using the project-level WEAI (pro-WEAI) and will also include modules on self-efficacy and decision-making with respect to health and nutrition. Maternal stress and wellbeing will be measured subjectively using validated scales such as Perceived Stress Scale (PSS). The interviews will also examine men’s knowledge and attitudes regarding the nutrition of women and children around the first 1000 days, women’s time and status and their productive and reproductive roles, fathers’ role in ensuring adequate health and nutrition of their family, and domestic violence.

#### Poultry value chain

Poultry VC-related data collected will include knowledge of improved poultry-production practices, input use and access to credit, poultry production and mortality, poultry marketing and transaction costs, and investments (e.g. housing).

#### Morbidity, hygiene, health and nutrition knowledge, and practices

Morbidity will be assessed through symptoms of acute respiratory infections, diarrhea, and fever in study children recalled through caregiver interviews over the last week and the last three days. Exposure to poultry feces and cleanliness of mother and child, kitchen, house, and compound will be measured by spot-check observations. Hygiene knowledge will be assessed using a questionnaire previously tested in Burkina Faso and practices will be measured by 24-h recall methods. The survey will include an assessment of women’s knowledge, attitudes, and practices regarding IYCF and sanitation and hygiene, using a questionnaire and 24-h recall methods previously tested in Burkina Faso [24].

#### Other household level data

The household survey will include a range of modules including demographics, consumption/expenditure, and income (gender-disaggregated; total income and income from sale of agricultural production); the consumption/expenditure module will also be used to derive household-level estimates of energy and micronutrient availability at the household level (and adequacy) using adult equivalents.

### Methods of analysis

#### Impact evaluation

The analysis will follow the intention-to-treat approach as protocol and as treated, using econometric analysis to control for differences at baseline across treatment arms for all the relevant variables of interest. The randomized study design allows for the identification of causal effects of interventions through comparisons of mean outcomes between the treatment arms. Following Bruhn and McKenzie [25], impact will be assessed for the different treatment arms using both a “difference-in-difference” (DID) estimator and a single difference analysis of covariance (ANCOVA) model. In particular, the ANCOVA estimator has been shown to provide more efficient estimates of program impact when auto-correlation of outcomes is low [25]. Depending on the level of clustering of the outcome under analysis, we will employ multi-level regression models that account for the hierarchical nature of the data [26]. The multi-level models, also known as mixed-effects models, will use both fixed effects (as covariates) and random effects at commune, village, and household levels to take into account the effect of clustering at these levels and to estimate unbiased standard errors. Robustness checks for the treatment estimates will also include controlling for the balancing variables used in the randomization. The intent-to-treat analysis strategy we will employ will include attempts to follow up all individuals in the randomized study, as well as the development of a main analysis that is valid under a stated plausible assumption on missing data, and sensitivity analysis to explore the effects of departures from the assumption underlying the main analysis. Data management, data cleaning, and statistical analyses will be conducted using Stata (Statacorp, USA). The statistical significance for all tests will be set at 5% in case of testing main effects or 10% in case of interactions. All statistical tests will be two-sided.

#### Market analysis

As far as we are aware, there are no published studies on poultry market integration in Burkina Faso. If poultry markets are not fully integrated, the additional supply and demand from the project could influence prices. There are several levels of markets along the poultry VC. These markets are linked by complex relations and involve several actors: producers, traders, aggregators, retailers, and consumers. The markets where the program may have an impact are the *foire* (village, or group of villages, level market), the aggregator market, and the retailer markets. *Foire* markets, in particular, are markets where foods are purchased from local farmers by traders and where local consumers make their purchases. If the project has an impact on prices it is likely to occur at this level. As part of the program monitoring activities, price data will be collected monthly in the local *foire* next to each of the selected villages. We will work with partners, including agriculture extension workers operating in the selected communes to use mobile phones to collect price monthly from relevant markets and from a sub-set of poultry producers.

#### Process evaluation and costs

The theory-driven process evaluation will combine qualitative and quantitative data to provide evidence of changes along the agriculture–nutrition pathways for the complex intervention package. We will work closely with the SELEVER implementers to identify indicators for the key processes and program impact pathways for the intervention. During the process evaluation and at end line, cost data will also be collected retrospectively following an ingredients approach using a semi-structured questionnaire. The questionnaire will also cover both cash and in-kind contributions and will be used to estimate both financial and economic costs. Financial costs capture actual expenditures in terms of program implementation on an annual basis. Economic costs included the opportunity costs of community members involved in the SELEVER service provision. Opportunity costs of volunteer community members will be calculated using local pay scales. Process and output data covering the adequacy of the service delivery will be collected using standardized data collection forms. Cost estimates will be combined with output data to provide estimates of cost-efficiency metrics and with impact estimates (at end line) to provide estimates of cost-outcomes.

#### Qualitative research

The qualitative research component will focus on understanding the impact pathways and the barriers related to social and cultural norms, especially for women. Gender-disaggregated individual interviews and focus group discussions will provide important information concerning how families make decisions about the production, sale, consumption, or purchase of nutritious foods. Qualitative interviews will also focus explicitly on women’s commitments and time allocation, on trade-offs they face, such as earning additional income vs. addressing domestic duties and caring for children, and whether engagement in these activities have the potential to improve women’s bargaining power.

### Data storage and security

Data cleaning will include the creation of anonymized versions of the datasets. The data files in Stata format containing identifying information from the baseline survey will be held only on the principal investigators’ (PI) computers, for use in tracking and identifying baseline survey respondents during the follow-up surveys. The data will be processed, cleaned, and documented by the research assistants and program manager in Burkina Faso, working under close guidance of the PIs. The documentation will include questionnaires, data variable labels, value labels, and a codebook, to assist in the analysis. Field notes from the fieldwork coordinator and team leaders describing the data collection process will also be stored. Within 12 months of the end of the project, the anonymized data will be made publicly available on the IFPRI web site. Users of the data will be asked to provide information on how the data are expected to be used, to help IFPRI track use of the data for research purposes.

### Dissemination of results

Our communications plan is focused on three objectives:

1. *Reaching the research community*. Making sure that our research is peer-reviewed, published, and widely cited by scholars in the nutrition, agriculture, and economics research communities is essential for augmenting the stock of knowledge on nutrition-sensitive agricultural interventions. We envisage our multidisciplinary research team publishing this research in the highest ranked field journals, but we will first focus on presenting preliminary research findings at international conferences and internal IFPRI seminars.
2. *Reaching the Burkinabe policy community*. Close engagement with government and civil society organizations in Burkina Faso will be a top priority for our communications plan. Our strategy will focus on regularly attending or co-organizing workshops in Ouagadougou to personally engage with local institutions, as well as research briefs for local dissemination.
3. *Reaching the international policy community.* Our unique focus on the hygiene dimensions of poultry systems has important implications for the international policy community. We will therefore place a high priority on reaching this community through a range of media (e.g. policy briefs, blogs, video interviews), close engagement with the CGIAR Agriculture for Nutrition and Health (A4NH) program’s communication team, and presentations of our findings in major IFPRI reports.

### Trial oversight

The evaluation will be guided by a steering group chaired by Marie Ruel, who is the Director of IFPRI’s Poverty, Health and Nutrition Division. The steering group will provide inputs and oversight at key stages of the proposed research. Additional steering group members include: Ministry of Health (Government of Burkina Faso), Harold Alderman (IFPRI), Agnes Quisumbing (IFPRI), John McDermott (A4NH), and Hannah Guedenet (ASI).

### Timeline

The timeline for the study is summarized in Fig. 2.

## Discussion

Low-income, rural populations subside on staple-based diets that generally lack the quality to meet food and nutrition requirements during key stages of the lifecycle [2]. Agricultural production is just one element within a food chain that affects the access, acceptability, and nutritional quality of foods for consumers. The VC framework, encompassing every activity from food production to consumption, can provide a useful lens to examine the role of interventions in food systems to improve diets [5]. However, the focus to date in VC analysis has been on economic returns, overlooking the opportunities to influence diets more broadly [6]. Moreover, VC activities may increase risks of exposure to toxins that may be harmful to health and nutrition. Poultry livestock rearing in low-income settings, in particular, may pose a risk to young children through a hypothesized oral–fecal pathway leading to EED [11]. However, there is a paucity of evidence on the effectiveness and additional risks involved in interventions in VCs to improve diets. To our knowledge, this study is the first to rigorously examine from a food systems perspective, the simultaneous impact of scaling-up nutrition-specific and nutrition-sensitive interventions through a livestock VC intervention platform, across nutrition, health, and agriculture domains. The findings of this evaluation will provide important evidence to support the design of market-based nutrition-sensitive interventions.

### Trial status

**Fig. 4:**
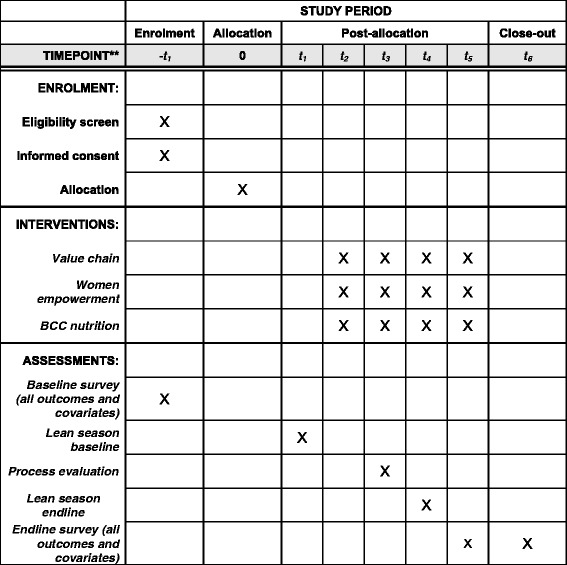
SPIRIT figure

Enrolment began in March 2017 (see SPIRIT figure [Fig. 4] and checklist [Additional file 1]).

## Additional file

Additional file 1: SPIRIT 2013 Checklist: recommended items to address in a clinical trial protocol and related documents. (DOC 120 kb)

## Abbreviations

A4NH: Agriculture for Nutrition and Health
ANCOVA: Analysis of covariance
ASI: Agribusiness Systems International
BCC: Behavior change communication
BMI: Body mass index
CGIAR: Consultative Group for International Agricultural Research
CRP: C-reactive protein
DD: Dietary diversity
DHS: Demographic Health Survey
EED: Environmental enteric dysfunction
HAD: Height for age deficit
HAZ: Height for age z-score
IFPRI: International Food Policy Research Institute
IYCF: Infant and young child feeding
MPA: Mean probability of adequacy
PA: Probability of adequacy
SELEVER: Soutenir l’Exploitation Familiale pour Lancer l’Élevage des Volailles et Valoriser l’Économie Rurale
TfR: Soluble transferrin receptor
VC: Value chain
VVV: Village volunteer vaccinators
WASH: Water, sanitation, and hygiene
WAZ: Weight for age z-score
WEAI: Women’s Empowerment in Agriculture Index
WHZ: Weight for height z-score
